# Involving forensic psychiatric patients in research - experiences, challenges and lessons learnt after one year of the PART Advisory Board

**DOI:** 10.1192/j.eurpsy.2025.1513

**Published:** 2025-08-26

**Authors:** E. Drewelow, M. Daum, K. Gerullis, I. Kilimann, O. Biernetzky, S. Teipel, P. Walde, B. Völlm

**Affiliations:** 1 Clinic for Forensic Psychiatry; 2 Clinic for Psychosomatic Medicine and Psychotherapy, Rostock University Medical Center; 3 Deutsches Zentrum für Neurodegenerative Erkrankungen (DZNE), Rostock; 4 LVR-Institut für Forschung und Bildung, Köln, Germany

## Abstract

**Introduction:**

Participatory research can enhance the relevance, quality, and impact of studies, increase recruitment, and optimize research methods. It can also help to secure third-party funding,. The participatory inclusion of forensic psychiatric patients in research has so far primarily been implemented in the UK. With the establishment of the PART advisory board, founded in December 2023, this gap is intended to be closed in Germany by creating sustainable, previously lacking structures for the involvement of forensic psychiatric patients. The acronym PART refers to participation/ participatory. In the PART advisory board, people with lived experience and researchers actively cooperate in projects in the field of forensic psychiatry. In preparation for their role, people with lived experience received training where they were provided with knowledge and skills for their work. The board has now advised several research projects and issued recommendations.

**Objectives:**

The purpose of this presentation is to critically reflect on the first experiences and consider how to incorporate these insights to improve the structure and working methods of the advisory board: What experiences have researchers, people with lived experience and those presenting their research projects made? How were participants involved? What impact did the advisory board have on specific research projects? What challenges arose, and how were they addressed? What are the lessons learned so far?

**Methods:**

To answer these questions, various evaluation tools were used. These include feedback questionnaires from participants assessing each advisory board meeting and project presentation, interviews with participants at least two times throughout the project, statements from the advisory board to project presenters, and responses from the presenters on how the board’s recommendations were implemented. Additionally, the perspectives of involved project staff were considered.

**Results:**

By the time interim results are presented at the EPA Congress 2025, the PART Forensic Advisory Board will have met at least 12 times and at least five research projects will have been presented to the board and received its recommendations. It has already become clear that involving participants, especially in forensic psychiatric settings, requires specific framework conditions. These address overarching structural aspects, disease specific patient relevant needs, as well as knowledge- and method-based issues relevant to participants.

**Conclusions:**

Even at this stage, the advisory board can be considered successfully implemented. Participatory research with people with lived experience in forensic psychiatric settings is possible, feasible, and above all, meaningful.

**Disclosure of Interest:**

None Declared

